# Regulation and function of the extracellular matrix protein tenascin-C in ovarian cancer cell lines

**DOI:** 10.1038/sj.bjc.6690410

**Published:** 1999-05

**Authors:** K E Wilson, J M S Bartlett, E P Miller, J F Smyth, P Mullen, W R Miller, S P Langdon

**Affiliations:** ICRF Medical Oncology Unit, Western General Hospital, Edinburgh, EH4 2XU, UK; Glasgow University Department of Surgery, Glasgow Royal Infirmary, Glasgow, G31 2ER, UK

**Keywords:** tenascin, ovarian cancer, TGF-β

## Abstract

The extracellular matrix glycoprotein tenascin-C (TN) is overexpressed in the stroma of malignant ovarian tumours particularly at the interface between epithelia and stroma leading to suggestions that it may be involved in the process of invasion (Wilson et al (1996) *Br J Cancer* **74**: 999–1004). To define regulation of TN further and investigate its function in ovarian cancer, a range of cell line models were studied. Concentrations of secreted TN in media from cultures of ovarian fibroblast cell lines were at least 100-fold greater than from carcinoma cell lines. Evidence for paracrine regulation of TN secretion was obtained by co-culture of carcinoma cells with fibroblast cells wherein secretion into the media was greater than from fibroblasts alone. Transforming growth factor (TGF)-β1, insulin-like growth factor (IGF)-II and progesterone all stimulated TN secretion while human choriogonadotropin (hCG), follicle-stimulating hormone (FSH) and γ-interferon inhibited secretion. TGF-β1 produced the greatest stimulation of TN in cultured fibroblasts and its co-expression with TN was examined in primary ovarian tumours. There was a significant association between the presence of moderate–strong expression of TN and TGF-β1. Evidence for TN having a functional role in ovarian carcinoma was obtained from adhesion and migration assays. The PE01, PE04, SKOV-3 and 59M cell lines all demonstrated marked adhesion to plastic coated with TN relative to the control protein bovine serum albumin (BSA) and expressed α2β1 and α3β1 integrins. The SKOV-3 cell line migrated more rapidly through TN than through BSA indicating that TN can facilitate migration of ovarian carcinoma cells. © 1999 Cancer Research Campaign


*British Journal of Cancer* (1999) **80**(5/6), 685–692
© 1999 Cancer Research Campaign
Article no. bjoc.1998.0410

### Regulation and function of the extracellular matrix protein tenascin-C in ovarian cancer cell lines

**KE Wilson[1], JMS Bartlett[2], EP Miller[1], JF Smyth[1], P Mullen[1], WR Miller[1] and SP Langdon[1]**

[1]ICRF Medical Oncology Unit, Western General Hospital, Edinburgh EH4 2XU, UK; [2]Glasgow University Department of Surgery, Glasgow Royal Infirmary, Glasgow G31 2ER, UK

**Summary** The extracellular matrix glycoprotein tenascin-C (TN) is overexpressed in the stroma of malignant ovarian tumours particularly at the interface between epithelia and stroma leading to suggestions that it may be involved in the process of invasion (Wilson et al (1996) *Br J Cancer* **74**: 999–1004). To define regulation of TN further and investigate its function in ovarian cancer, a range of cell line models were studied. Concentrations of secreted TN in media from cultures of ovarian fibroblast cell lines were at least 100-fold greater than from carcinoma cell lines. Evidence for paracrine regulation of TN secretion was obtained by co-culture of carcinoma cells with fibroblast cells wherein secretion into the media was greater than from fibroblasts alone. Transforming growth factor (TGF)-β1, insulin-like growth factor (IGF)-II and progesterone all stimulated TN secretion while human choriogonadotropin (hCG), follicle-stimulating hormone (FSH) and γ-interferon inhibited secretion. TGF-β1 produced the greatest stimulation of TN in cultured fibroblasts and its co-expression with TN was examined in primary ovarian tumours. There was a significant association between the presence of moderate–strong expression of TN and TGF-β1. Evidence for TN having a functional role in ovarian carcinoma was obtained from adhesion and migration assays. The PE01, PE04, SKOV-3 and 59M cell lines all demonstrated marked adhesion to plastic coated with TN relative to the control protein bovine serum albumin (BSA) and expressed α2β1 and α3β1 integrins. The SKOV-3 cell line migrated more rapidly through TN than through BSA indicating that TN can facilitate migration of ovarian carcinoma cells.

**Keywords:** tenascin; ovarian cancer; TGF-β

Tenascin-C (TN) is a large hexameric glycoprotein found in the extracellular matrix (Erickson and Bourden, 1989; Chiquet-Ehrismann, 1993). It is thought to be involved in numerous cellular functions including adhesion, migration, embryonic development, wound healing and tumour metastasis (Erickson and Bourden, 1989; Chiquet-Ehrismann, 1993). It is strongly expressed in developing fetal tissues, while in normal adult tissues and organs its expression is generally limited to areas associated with proliferation and cellular re-organization. In many solid tumours, TN is overexpressed in the stroma and this led to the suggestion that it may be associated with malignant invasion (Mackie et al, 1987). Several forms of TN protein are generated by alternative splicing of one common primary transcript (Chiquet-Ehrismann, 1993) and we have previously identified multiple RNA transcripts in malignant ovarian tumours (Wilson et al, 1996). We have also demonstrated that TN is overexpressed in the stroma of malignant ovarian tumours at a significantly greater incidence and intensity than found in benign ovarian tumours (Wilson et al, 1996). The highest level of expression is observed at epithelial–mesenchymal junctions, leading us to speculate on the cellular source of TN, and the presence of paracrine regulation of TN secretion (Wilson et al, 1996). The transient and dynamic occurrence of TN in normal cellular systems has led to suggestions of it being regulated by growth factors and cytokines and several inducers, most notably transforming growth factor (TGF)-β, have been identified (Pearson et al, 1988; Rettig et al, 1994).

In this study we have investigated the production of TN in epithelial and stromal ovarian cell lines to determine the primary cell type producing TN and have used a co-culture system to identify whether paracrine influences might be involved in its regulation. The effects on TN regulation of specific growth factors, cytokines and hormones which are likely to be present in vivo were then investigated. Finally evidence for a functional role in invasion was sought by the use of adhesion and migration assays and the expression of several integrins known to bind TN was examined.

### MATERIALS AND METHODS

### Cell lines

The human ovarian carcinoma cell lines PE01, PE04 and PE01$^{CDDP}$ were established and characterized as previously described (Langdon et al, 1988). The SKOV-3 and 59M ovarian carcinoma cell lines were obtained from the European Collection of Animal Cell Cultures, Porton Down, UK. All these lines were routinely cultured at 37°C in an atmosphere of 5% carbon dioxide/95% air in Dulbecco's modified Eagle medium (DMEM) containing phenol red indicator. The medium was supplemented with 10% fetal calf serum (FCS), 2 mM L-glutamine, 100 IU ml$^{-1}$ penicillin and 100 μg ml$^{-1}$ streptomycin.

The fibroblast cell lines PE012F, PE027F, PE09F and PE013F were initiated from ascitic cells obtained from patients with primary ovarian cancer. Ascitic cells which had been washed in

*Received 11 August 1998*
*Revised 22 December 1998*
*Accepted 23 December 1998*

*Correspondence to:* SP Langdon

**Table 1** Co-expression of TN and TGF-β in a series of malignant ovarian tumours

| Sample | TN protein | TGF-β–RNA isoform expression | | |
|---|---|---|---|---|
| | | TGF-$\beta_1$ | TGF-$\beta_2$ | TGF-$\beta_3$ |
| 002 | +++[a] | +[b] | + | + |
| 006 | – | + | – | + |
| 008 | + | + | – | + |
| 014 | ++ | + | + | + |
| 015 | + | – | – | + |
| 019 | +++ | + | – | – |
| 020 | – | – | + | – |
| 021 | ++ | + | + | – |
| 069 | +++ | + | + | + |
| 072 | ++ | + | + | – |
| 077 | ++ | + | + | + |
| 095 | +++ | – | + | – |
| 096 | – | – | + | – |
| 104 | – | – | + | + |
| 109 | + | – | + | + |
| 117 | + | + | + | – |
| 122 | ++ | + | + | – |
| 124 | ++ | + | + | + |
| 132 | +++ | + | + | + |
| 134 | – | + | – | – |
| 137 | + | – | + | – |
| 148 | + | – | – | – |
| 149 | ++ | – | + | – |

[a]TN protein: – = negative; + = weak staining; ++ = moderate staining; +++ = strong staining. [b]TGF-β RNA isoform: + = positive; – = negative.

**Table 2** Percentage migration of ovarian cancer cell lines through TN and FN

| | Migration (%) | | |
|---|---|---|---|
| Cell line | BSA control | TN | FN |
| SKOV-3 | 2.8 (0.3[a)] | 19.2 (4.9) | 25.3 (2.1) |
| PE01 | 3.5 (1.3) | 8.1 (0.9) | 5.0 (0.2) |

[a]The percentage of cells migrating through the ECM matrix in a 48 h period is shown. Each value is the mean ± standard deviation of triplicate wells. TN = tenascin-*C*;FN = fibronectin; BSA = bovine serum albumin.

DMEM + 10% FCS were placed into 25-cm$^2$ flasks and allowed to adhere. The fibroblast cells were selected on the basis of their rapid attachment to tissue culture plastics. Fibroblasts attached to the plastic substrate more rapidly than epithelial cells and media containing unattached cells were poured off after 2–4 h, leaving a predominantly fibroblastic population.

The original ascites preparations contained a high percentage of leucocytes and epithelial cells with a minority (approximately 10%) of fibroblasts. After the above culture selection, the final populations of fibroblasts were found to be > 99% pure with less than 1% contamination by leucocytes or tumour cells as indicated by immunocytochemistry.

### Immunocytochemistry

Once the cultures were established, after 1–2 passages, multispot slides were prepared and immunocytochemistry was used to compare the samples of the fibroblast cultures with the original ascites population. Cells were incubated with either 5B5 mouse anti-human fibroblast monoclonal antibody (used at 1:100; Dako, Ely, UK), 2B11 mouse anti-human leucocyte common antigen (used at 1:10; Dako, Ely, UK), E29 mouse anti-human epithelial membrane antigen (used at 1:40; Dako, Ely, UK) or Tris-buffered saline (TBS: as control) for 30 min at room temperature. After washing in TBS, multispots were treated with rabbit anti-mouse biotinylated antibody diluted 1:100 in TBS, followed by avidin–biotin peroxidase complex made up in TBS; both incubations being for 30 min at room temperature and followed by washing in TBS. After the final wash, multispots were treated with a solution of 3,3′-diaminobenzidine (1 mg ml$^{-1}$) containing 5% hydrogen peroxide for 5 min. Multispots were then dehydrated, cleared and mounted under coverslips with DPX mounting medium.

### Preparation of cells for measurement of TN secretion

Cells were plated onto tissue culture flasks or trays at high density (70–90% confluence) in DMEM (+ 10% FCS). The serum-containing medium was then removed and the cells were washed in phosphate-buffered saline (PBS) before the addition of serum-free medium, phenol red-free DMEM containing HITS (10 nM hydrocortisone, 5 μg ml$^{-1}$ insulin, 10 μg ml$^{-1}$ transferrin and 30 nM sodium selenite). After a wash-out period (~12 h), fresh media were added and the cells were incubated for 48 h. After this time, the conditioned medium (CM) was collected and centrifuged in polypropylene tubes at 2000 rpm to remove dead cells and other debris. The cells were then harvested and counted. CM was immediately plated out for an enzyme-linked immunosorbent assay (ELISA) as described below.

To assess the effects of growth factors, hormones and cytokines on TN secretion, concentrations were selected which had previously been determined to produce effects on growth in ovarian systems. These factors were added to the cells in a total volume of 500 μl of medium. The cells were incubated for 48 h, counted and the medium assayed for TN content by ELISA as described below.

### ELISA

Some 96-well plastic plates (Immulon 4) were coated overnight with purified TN (Gibco BRL, Paisley, UK) diluted in PBS (200 μl) to give a range of 0.5–16 ng TN per well for a standard curve. Each sample of CM (200 μl), was also added to wells for overnight incubation. CM was assessed neat and diluted 1:2 with PBS. Following the overnight incubation the plates were thoroughly washed with PBS containing 0.5% Tween-20 (PBS-T). Half the plate was then incubated for 2 h at 37°C with monoclonal mouse anti-human TN antibody (Dako, Ely, UK) at a 1:160 dilution in PBS-T while the remaining half was left in PBS-T as a measure of background binding. The plates were washed and incubated with rabbit anti-mouse Ig diluted 1:1000 in PBS-T for 1 h at 37°C before a final washing in PBS-T. A total of 50 mg of orthophenylenediamine (OPD) were dissolved in 100 ml of substrate buffer (0.71 g anhydrous $Na_2HPO_4$, 0.5 g citric acid, pH 5.0). Immediately prior to use, 20 μl of hydrogen peroxide were added. OPD solution (200 μl) was added to each well and the plate was incubated for 10 min at room temperature in the dark. The reaction was terminated by addition of 50 μl of 0.5 M sulphuric acid and the optical density of each well was measured on a spectrophotometer at a wavelength of 492 nm.

A 1:2 dilution was also routinely assayed for all samples and these were shown to dilute in similar fashion to TN standards giving an absorbance value half that of neat samples. The numbers

 © Cancer Research Campaign 1999

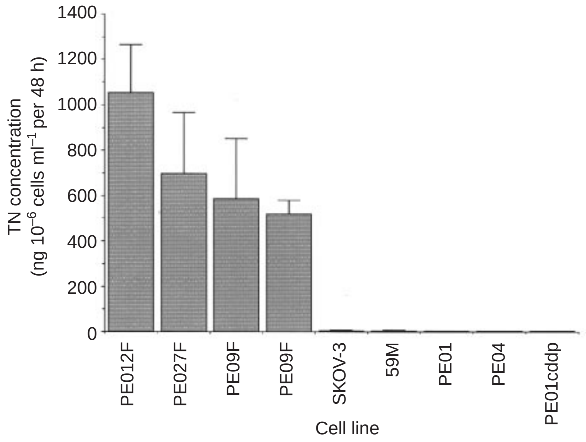

**Figure 1** Levels of TN in media from ovarian fibroblast and carcinoma cell lines. PE012F, PE027F, PE09F and PE013F are fibroblast lines and PE01, PE04, PE01$^{CDDP}$, SKOV-3 and 59M are carcinoma lines. Mean values standard error from a typical assay are shown. Cells were incubated in serum-free medium for 48 h and TN content was measured by ELISA as described in Materials and Methods of cells secreting TN into the media were counted and the measurements of TN secretion were corrected for cell number. Within each assay duplicate samples were measured and the secretion of TN was expressed as the ng produced in the 48-h incubation period per million cells per ml of media (ng of TN per $10^6$ cells $ml^{-1}$ per 48 h). Each cell line was assayed on a minimum of two separate occasions and the mean secretion of TN was calculated.

### Co-culture experiments

Tissue culture inserts with 8-μm-diameter pores were used to co-culture fibroblast and epithelial cells. Fibroblasts ($2 \times 10^5$) were added to the well and a similar number of PE01 epithelial cells were added to the inserts (in a separate tray). The cells were allowed to attach for 24 h in DMEM (+10% FCS) and incubated overnight in DMEM (+ HITS). After this time the inserts were moved to the tray containing the fibroblasts. Fresh serum-free medium was added to the well and the insert (500 μl and 300 μl respectively). The cells were incubated for 48 h at 37°C and counted; the media were collected and assayed for TN content by ELISA.

### Cell adhesion assay

Non-sterile 96-well trays were coated overnight at 4°C (or for 2 h at 37°C) with the extracellular matrix proteins (10 μg $ml^{-1}$) TN, fibronectin (Sigma, Poole, UK) or collagen IV (Sigma, Poole, UK) in quadruplicate wells. To take into account non-specific binding to protein 1% bovine serum albumin (BSA) solution (in PBS) was used as a control. After coating, the plates were washed in PBS. Non-specific adherence to plastic was blocked by incubation for 90 min in 0.1% BSA solution. Cells were harvested in a small amount of trypsin to produce a single cell population, and labelled with chromium ($^{51}Cr$) as described by Brunner et al (1976). The cells were washed three times in serum-free media to remove traces of extracellular matrix (ECM) proteins and serum, and resuspended to produce a concentration of $3 \times 10^5$ per ml. Aliquots (50 μl) of the suspension were added to each well and to counting tubes as the 'input' count. After incubation at 37°C for 2 h, plates were washed twice by gently submerging the plate in PBS supplemented by cations (1 mM $Ca^{2+}$/0.5 mM $Mg^{2+}$). Plates were then cut into individual wells and counted in a γ-counter.

### Cell migration assay

Migration assays were performed in 8-μM-diameter pore size Transwell chambers in a method adapted from Mould et al (1994). The undersurface of the polycarbonate membrane was coated with ECM proteins by placing the insert into the relevant protein solution (TN, fibronectin or BSA; 10 μg $ml^{-1}$) for 1 h at 37°C. The protein solution was removed and the membranes were washed in PBS. Cells were harvested to produce a single-cell suspension of $4 \times 10^5$ cells $ml^{-1}$ in serum-free DMEM containing 1% BSA. Aliquots of the cell suspension ($1 \times 10^5$) were added to the upper chamber of the Transwell and 500 μl of the same media were added to the lower chamber. The cells were incubated at 37°C for 48 h in a humidified incubator and cells allowed to migrate. Cells were then harvested by trypsinization and migration was expressed as the percentage of cells that had passed through the membrane.

### Analysis of integrin expression by flow cytometry

Cells were harvested using trypsin–EDTA which was immediately neutralized with DMEM containing 10% FCS. Cells were then collected after centrifugation at 2000 rpm for 5 min and resuspended in complete medium at $1 \times 10^6$ cells $ml^{-1}$ and kept in an incubator at 37°C for 30 min before staining. For each cell line, 3 aliquots of $5 \times 10^5$ cells were incubated with each primary antibody. The following monoclonal antibodies were used: JB1 (anti-β1 integrin; Chemicon, Harrow, UK), P1E6 (anti-α2β1; Dako, Ely, UK), P1B5 (anti-α3β1; Dako, Ely, UK), LM609 (anti-αvβ3; Chemicon, Harrow, UK) and anti-annexin II (Affiniti, Mamhead, UK). The aliquots were washed once in ice-cold PBS, then in PBS containing 5% FCS before addition of 100 μl of diluted antibody (1:50 for JB1 and LM609; 1:40 for P1E6 and P1B5 and 1:20 for annexin II) to each tube. Negative controls had 100 μl PBS–FCS added. The tubes were incubated on ice for 60 min, then washed once in PBS–FCS. Rabbit anti-mouse immunoglobulin RPE conjugate (100 μl) was added to each tube and incubated on ice for 60 min. The cells were again washed in PBS–FCS and resuspended in 1 ml PBS for analysis on a FACScan flow cytometer using the Lysys II program.

### RESULTS

### Secretion of TN by ovarian cell lines

To identify the major ovarian cell type (fibroblast or epithelial) producing TN, conditioned media were collected from ovarian fibroblast and ovarian carcinoma cell lines cultured under serum-free conditions for 48 h and assayed by ELISA. The concentrations of TN in media from the fibroblast cell lines PE09F, PEO12F, PE013F and PE027F ranged from 519 to 1053 ng TN $10^{-6}$ cells $ml^{-1}$ per 48 h (Figure 1). In contrast, the concentrations in the media from the ovarian carcinoma cell lines were < 1% of the fibroblast levels with SKOV-3 and 59M producing approximately 4 ng $10^{-6}$ cells $ml^{-1}$ per 48 h, while media from the remaining three carcinoma cell lines PE01, PE04 and PE01$^{CDDP}$ did not contain measurable TN (< 2 ng $10^{-6}$ cells $ml^{-1}$ per 48 h).

*© Cancer Research Campaign 1999*

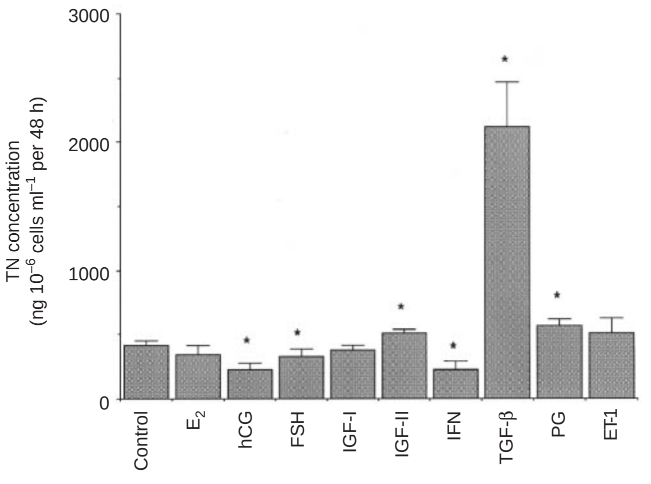

**Figure 2** Modulation of TN concentration in media conditioned by the PE012F fibroblast cell line after exposure to hormones, growth factors and cytokines. These data represent the mean values of TN secretion from triplicate wells with error bars representing standard deviation. The concentrations used were as follows: $E_2$ (17-β-oestradiol), 0.1 nM; hCG (human choriogonadotropin), 10 IU $ml^{-1}$; FSH (follicle-stimulating hormone), 0.1 IU $ml^{-1}$; IGF-I (insulin-like growth factor I), 10 nM; IGF-II (insulin-like growth factor II), 10 nM; IFN (interferon-γ), 10 IU $ml^{-1}$; TGF-β (transforming growth factor β1), 2.4 nM; PG (progesterone), 0.1 nM; ET-1 (endothelin-1), 10 nM. Cells were incubated in serum-free medium containing the above factors for 48 h and TN content was measured by ELISA as described in Materials and Methods. *$P < 0.05$ significantly different from the control (Student's *t*-test)

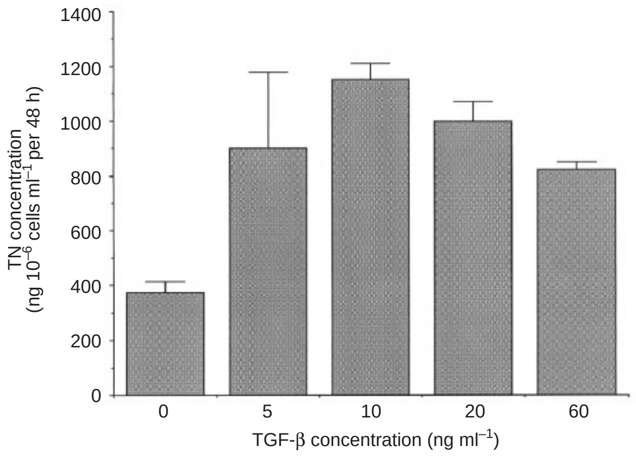

**Figure 3** Modulation of TN concentration in media conditioned by the PE012F fibroblast cell line after exposure to varying concentrations of TGF-β1 (transforming growth factor $β_1$). The values shown are mean values from quadruplicate wells and error bars represent standard deviations. Cells were incubated in serum-free medium containing TGF-$β_1$ for 48 h and TN content was measured by ELISA as described in Materials and Methods

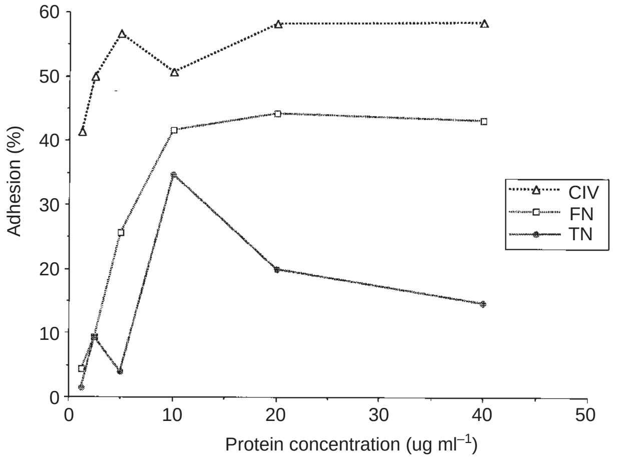

**Figure 4** Attachment of SKOV-3 ovarian cancer cells to varying concentrations of ECM proteins. These data represent the percentage of cells binding to collagen IV (CIV), fibronectin (FN) and tenascin (TN) after 2 h. Data shown represent mean values from quadruplicate wells. Error bars are not shown, but were always < 10% of the mean values

### Modulation of TN secretion

Observations of primary tumours suggested that the strongest expression of TN was at junctions between carcinoma cells and stroma, implying possible paracrine regulation. In order to investigate this in a tissue culture model, ovarian fibroblasts (PE012F or PE09F) were co-cultured with the PE01 ovarian carcinoma cell line. The fibroblast and epithelial cell populations were kept separate using porous tissue culture inserts with the fibroblasts being placed at the bottom of the well and PE01 carcinoma cells being grown in the insert.

Media were collected from the cells after 48 h of co-culture and assayed for TN content by ELISA. PE01 cells generated a level of < 2 ng TN $10^{-6}$ cells $ml^{-1}$ per 48 h, while PE012F and PE09F cells produced concentrations of 342 and 98 ng TN $10^{-6}$ cells $ml^{-1}$ per 48 h, respectively, when the lines were grown in the absence of the other cell type. When the fibroblasts were co-cultured with the PE01 cells, the level of TN measured increased to 432 and 131 ng TN $10^{-6}$ cells $ml^{-1}$ per 48 h for the PE012F–PE01 and PE09F–PE01 co-cultures respectively. Repetition of this experiment indicated a small, but consistent, 20–30% increase in TN secretion by co-culture. This increase in TN secretion was observed on 11 of 12 occasions ($P = 0.0034$, Wilcoxon signed rank test).

### Effects of growth factors, hormones and cytokines on TN secretion

The effects of specific growth factors, hormones and cytokines as regulators of TN expression were investigated as these might mediate the paracrine interaction between fibroblast and epithelial cells. The agents examined were chosen on the basis of their modulation of TN secretion in other cell types and also on their effects on ovarian cell behaviour. The concentrations used for the initial screening had previously been established to produce growth effects in ovarian cancer cells. All these factors were tested for cross-reactivity in the ELISA and found to be negative. Figure 2 illustrates the modulation of TN secretion in the PE012F ovarian fibroblast cell line by this range of factors. TN secretion in the PE012F ovarian fibroblast cell line was markedly stimulated by TGF-$β_1$ (2.4 nM). While insulin-like growth factor (IGF)-II (10 nM) and progesterone (0.1 nM) did not produce a large increase, they consistently stimulated TN secretion above control levels. Conversely, TN secretion was inhibited by the gonadotropins, human choriogonadotropin (hCG; 10 IU $ml^{-1}$) and follicle-stimulating hormone (FSH; 0.1 IU $ml^{-1}$) and also γ-interferon (10 IU $ml^{-1}$). HCG produced the greatest effect decreasing TN secretion to approximately 50% of control levels. 17β-Oestradiol (0.1 nM), IGF-I (10 nM) and endothelin-1 (10 nM)

© Cancer Research Campaign 1999

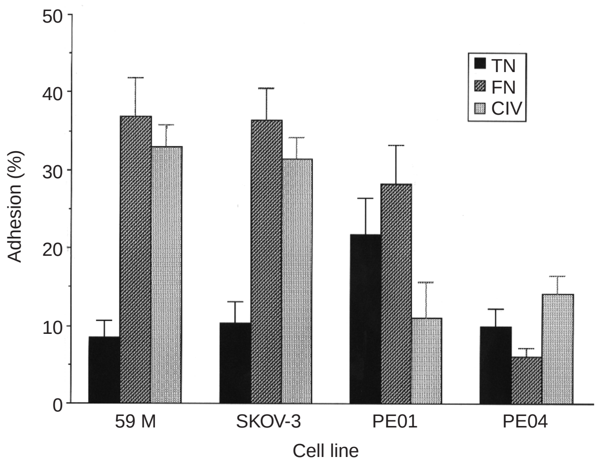

**Figure 5** Attachment of ovarian cancer cells to different ECM proteins. The percentage attachment after 2 h is recorded and values shown are mean values from quadruplicate wells and the error bars represent standard deviation. The ECM proteins (TN = tenascin; FN = fibronectin; CIV = collagen IV) were used at a concentration of 10 μg ml$^{-1}$

had no effect on TN secretion. TGF-$\beta_1$ produced the largest stimulation of TN secretion of all the factors tested. To investigate whether TGF-$\beta_1$ exerted its effects in a dose-dependent manner the factor was added in a range of concentrations, as used by Pearson et al (1988). Figure 3 illustrates the modulation of TN secretion in PE012F fibroblasts by TGF-$\beta_1$. All concentrations tested between 5 and 60 ng ml$^{-1}$ enhanced TN secretion, with maximum secretion being observed at 10 ng ml$^{-1}$.

### Tenascin expression and TGF-β isoform expression in primary ovarian tumours

To examine the association between TN and TGF-β expression further, the incidence of co-expression was explored in a series of 23 primary ovarian tumours. TN expression had previously been reported in a cohort of ovarian carcinomas (Wilson et al, 1996) and, in a separate study, the presence of different TGF-β isoforms had been defined (Bartlett et al, 1997). The expression of both TN and TGF-β isoforms is shown in Table 1. Of the 23 tumours examined, 14 expressed TGF-$\beta_1$ mRNA, 17 expressed TGF-$\beta_2$ mRNA and 13 expressed TGF-$\beta_3$ mRNA. If tumours are divided into those expressing moderate–strong levels of TN are compared with those expressing weak–no staining then analyses using Fischer's exact test indicated a significant relationship ($P$ = 0.036) between TGF-$\beta_1$ expression and TN, but not between TGF-$\beta_2$ or TGF-$\beta_3$ and TN ($P$ = 0.069 and 1.00 respectively).

### Effect of TN on ovarian cancer cell adhesion and migration

The adhesion of the ovarian carcinoma cell lines to TN, fibronectin and collagen IV was investigated. BSA (1%) was used as a control to examine any non-specific protein binding; the binding to BSA was always less than 10% of the input value. An initial experiment using the SKOV-3 cell line determined the optimal concentration of ECM protein. For all three ECM proteins, 10 μg ml$^{-1}$ produced maximum or near maximum binding and was therefore selected for subsequent experiments (Figure 4). While concentrations of 20 and 40 μg ml$^{-1}$ produced effects similar to that obtained at 10 μg ml$^{-1}$ for both collagen IV and fibronectin, the adhesion for TN showed a biphasic pattern (Figure 4).

The adhesion of the 59M, PE01 and PE04 cell lines to the ECM proteins (in addition to the SKOV-3 cell line) are shown in Figure 5. The SKOV-3 and 59M cell lines show comparable levels of adhesion to each other; in the wells containing fibronectin and collagen IV, 30–40% of the cells attached within 2 h. In the wells containing TN, attachment was approximately 10% of the input value. The PE01 cell line showed a different preference for the ECM proteins. The greatest level of adhesion (29%) was observed on fibronectin with only a slightly reduced level of binding to TN (22%). The poorest substrate for adherence of PE01 cells was collagen IV, which demonstrated only 11% adhesion. PE04 cells, overall, demonstrated the lowest levels of adhesion, with maximum levels of 14% seen on collagen IV, 10% binding for TN and the least preference for fibronectin (6%). All these values were corrected for non-specific protein binding by subtracting the level of adhesion to the wells containing BSA. Therefore, it can be seen from these data that TN does promote the attachment of ovarian carcinoma cells; however, different cell lines have varying affinities for the ECM proteins studied.

The migration of SKOV-3 and PE01 cells through TN was compared with migration through BSA and fibronectin. Cells were allowed to migrate for 48 h through a porous transwell insert whose underside was coated with each respective protein and migration assessed as the percentage of cells passing through the membrane (Table 2). For SKOV-3 cells, while only 2.8% of cells migrated through BSA, 19.2% migrated through TN and 25.3% through fibronectin indicating that both proteins promote migration of this cell line. For PE01 cells, migration was only slightly increased in tenascin (8.1% migration) and even less so in fibronectin (5% migration) compared to the BSA control (3.5% migration) (Table 2).

### Integrin profiles of the cell lines

The expression of several integrins that are known to bind TN was investigated. $\beta_1$ receptors were identified in the 59M, SKOV-3, PE01 and PE04 cell lines and specific antibodies for the $\alpha_2\beta_1$ and $\alpha_3\beta_1$ integrins indicated expression of both integrins in all four cell lines (Figure 6). The $\alpha_v\beta_3$ integrin was found at low levels in the 59M and SKOV-3 lines but not in the PE01 or PE04 lines (Figure 6). Cell surface annexin-II expression was not detected in any of these lines.

### DISCUSSION

In the present study, measurement of TN by ELISA demonstrated that media from ovarian fibroblasts contained TN at levels approximately 100-fold higher than from ovarian carcinoma cell lines. This is consistent with the strong stromal staining of TN found in primary ovarian carcinoma sections and suggests that fibroblasts are likely to be the primary source of TN. Several other studies have demonstrated that fibroblasts are capable of producing TN and the levels in this study are comparable with levels reported for non-ovarian fibroblasts (Erickson and Bourd0n, 1989). The ovarian carcinoma cell lines SKOV-3 and 59M also produced low levels of TN and these are the first data reported for ovarian carcinoma lines.

© Cancer Research Campaign 1999

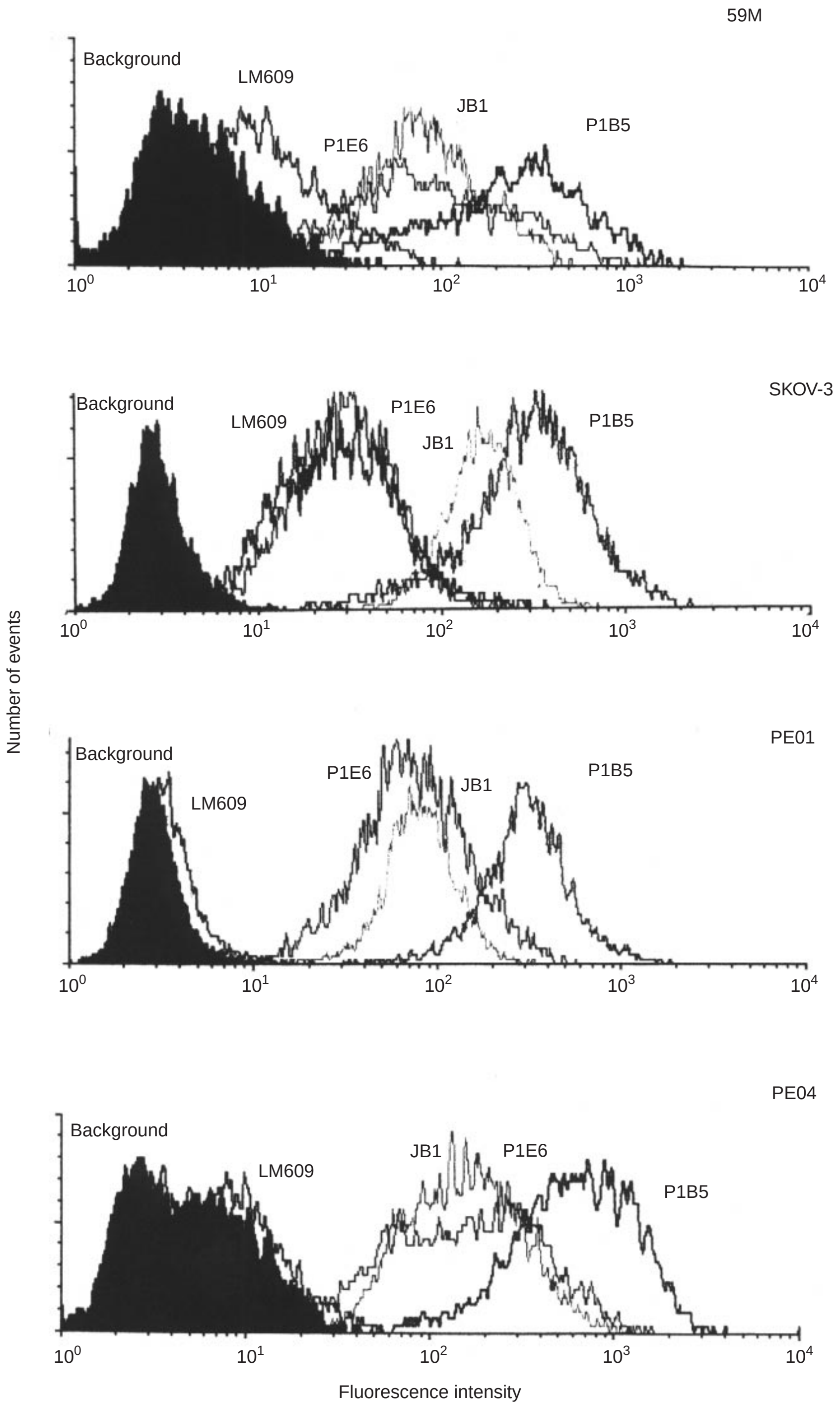

**Figure 6** Integrin expression in ovarian carcinoma cell lines. Cells were incubated with anti-integrin antibodies for 60 min on ice and then incubated with rabbit anti-mouse immunoglobulin RPE conjugate for 60 min. Cells were then analysed on a FACScan flow cytometer. Values are expressed as fluorescence intensity against number of events. The following monoclonal antibodies were used: JB1 (anti-$\beta_1$ integrin), P1E6 (anti-$\alpha_2\beta_1$), P1B5 (anti-$\alpha_3\beta_1$) and LM609 (anti-$\alpha_v\beta_3$). The background profile refers to the fluorescence produced in the absence of primary antibody. The profile for annexin-II was identical to that of the background

In a previous study (Wilson et al, 1996) we observed that TN was not only overexpressed in the stroma of primary ovarian cancers but this expression was most intense at the interface between carcinoma cells and stroma suggestive of paracrine regulation. Co-culture of ovarian fibroblasts with ovarian carcinoma cells led to an increased secretion of TN and this would be consistent with observations for breast cancer wherein media conditioned by MCF-7 breast carcinoma cells induced fibroblasts to synthesize TN (Chiquet-Ehrismann et al, 1989). In this latter study, the inducer within the conditioned medium was shown to be TGF-$\beta_1$. Because of this we have studied the effects of TGF-β and a number of important ovarian regulators and products for their ability to modulate TN

*© Cancer Research Campaign 1999*

secretion. Of these, TGF-$\beta_1$ proved to be the strongest inducer in our ovarian fibroblast model. Further support that this peptide growth factor may be an important regulator of TN in ovarian cancer was obtained from an analysis of primary ovarian carcinoma sections, which indicated that TN expression was significantly associated with TGF-$\beta_1$ expression. A similar association has been reported in invasive breast carcinoma (Walker et al, 1994) and in the tissue distribution in mouse embryos (Pearson et al, 1988).

While TGF-β appears to be an important, and perhaps even principal, regulator of TN, there are data to indicate that a number of other extrinsic factors may also contribute jointly to the regulation of TN secretion in a modular fashion (Rettig et al, 1994). These include epithelial growth factor (EGF), fibroblast growth factor (FGF), interleukin (IL)-1, IL-4, tumour necrosis factor (TNF)-α, platelet derived growth factor (PDGF), steroid hormones, glucocorticoids and angiotensin II. Of the agents investigated that have important roles in normal ovarian physiology, progesterone and IGF-II produced small but consistent stimulations, while the gonodotrophins FSH and hCG (which produces the same biological responses as luteinizing hormone) and γ-interferon decreased TN secretion. It seems likely that in vivo, these separate processes may well be integrated to produce a final response. In addition to these soluble factors, it is also likely that cell–cell contacts will be important in regulating secretion of TN.

During malignant invasion and metastasis the processes of both adhesion and anti-adhesion are important. TN has been shown to contain both adhesive (within its alternatively spliced region) and anti-adhesive elements (within the EGF-like repeat structures) (Spring et al, 1989). In the present study, TN enhanced the adhesion of all four ovarian carcinoma cell lines examined compared to the BSA control and this may be important in promoting attachment to the basement membrane prior to proteolysis and facilitating migration through the ECM. These cell lines also adhere as well, if not better, to fibronectin and an alternative mode of action of TN is in its competition with fibronectin for cell binding thereby inhibiting integrin-mediated attachment of cells to fibronectin and allowing cell movement to take place (Chiquet-Ehrismann et al, 1988). Furthermore, TN can physically bind to fibronectin and sterically blocking cell access to the matrix (Chiquet-Ehrismann et al, 1988). An intriguing observation using a glioma model has recently reported wherein TN was found to provide a positive effect on fibronectin-mediated migration by altering cell morphology and enhancing cell motility (Deryugina and Bourdon, 1996).

A biphasic effect similar to that observed for SKOV-3 cells binding to TN has been previously observed for glioma cell attachment by Giese et al (1996). These investigators observed maximum binding of glioma cells at 10 μg $ml^{-1}$ but decreased binding at 33 and 100 μg $ml^{-1}$. Based on this observation together with data from migration and integrin analysis studies, they concluded that glioma cells express two separate receptors which allow these opposing effects to occur at different densities of TN; such a view might explain these observations in SKOV-3 cells. TN also promoted the migration of the SKOV-3 and PE01 cell lines. This promotion of migration may allow ovarian carcinoma cells to invade more readily into surrounding stroma. In other systems TN can both promote (Deryugina and Bourdon, 1996) or inhibit (Hoffman et al, 1994) cellular migration.

TN is known to bind to several integrins on the cell surface, including $\alpha_2\beta_1$, $\alpha_8\beta_1$, $\alpha_9\beta_1$, $\alpha_v\beta_3$, $\alpha_v\beta_6$ and annexin-II (Yokasaki and Sheppard, 1995; Chung and Erickson, 1994). Antibodies to several of these molecules were available, and $\beta_1$ and specifically $\alpha_2\beta_1$ and $\alpha_3\beta_1$ expression were found in all four cell lines examined. $\alpha_v\beta_3$ was expressed in two of the four lines, while annexin-II was not detected in any of these lines. Cell adhesion to TN in several normal and malignant cell types has previously been shown to involve at least two sites, the SRRGDMS site which binds $\alpha_v\beta_3$ and $\alpha_v\beta_6$, and a non-RGD site which binds $\alpha_2\beta_1$ (Deryugina and Bourdon, 1996). The $\alpha_2\beta_1$ site has been proposed to play a more important role in TN cell migration (Deryugina and Bourdon, 1996). The $\alpha_v\beta_3$ integrin is involved in the migration of fibronectin and the higher expression of $\alpha_v\beta_3$ and lower expression of $\alpha_2\beta_1$ in SKOV-3 cells compared to PE01 cells may contribute to the more rapid migration of SKOV-3 cells through fibronectin than TN while PE01 cells migrate more rapidly through TN than fibronectin.

In conclusion, these data indicate that in ovarian cancer systems, TN is secreted predominantly from fibroblasts, is regulated by a variety of factors including TGF-β and has effects on both cell adhesion and migration suggesting a facilitating role in invasion.

### REFERENCES

Bartlett JMS, Langdon SP, Scott WN, Love SB, Miller EP, Katsaros D, Smyth JF and Miller WR (1997) Transforming growth factor beta isoform expression in human ovarian tumors. *Eur J Cancer* **33**: 2397–2403

Brunner KT, Enger HDF and Cerottini JC (1976) The $^{51}Cr$ release assay as used for the quantitative measurement of cell mediated cytolysis in vivo. In *In Vitro Methods in Cell-Mediated and Tumour Immunity*. Bloom BR and Davids JHR (eds), pp. 423–436. Academic Press: New York

Chiquet-Ehrismann R (1993) Tenascin and other adhesion-modulating proteins in cancer. *Semin Cancer Biol* **4**: 301–310

Chiquet-Ehrismann R, Kalla P, Pearson C, Beck K and Chiquet M (1988) Tenascin interferes with fibronectin action. *Cell* **53**: 383–390

Chiquet-Ehrismann R, Kalla P and Pearson CA (1989) Participation of tenascin and transforming growth factor-β in reciprocal epithelial-mesenchymal interactions of MCF7 cells and fibroblasts. *Cancer Res* **49**: 4322–4325

Chung CY and Erickson HP (1994) Cell surface annexin-II is a high affinity receptor for the alternatively spliced segment of tenascin-C. *J Cell Biol* **126**: 539–548

Deryugina EI and Bourdon MA (1996) Tenascin mediates human glioma cell migration and modulates cell migration on fibronectin. *J Cell Sci* **109**: 643–652

Erickson H and Bourdon M (1989) Tenascin: an extracellular matrix protein prominent in specialized embryonic tissues and tumours. *Ann Rev Cell Biol* **5**: 71–92

Giese A, Loo MA, Norman SA, Treasurywala S and Berens ME (1996) Contrasting migratory response of astrocytoma cells to tenascin mediated by different integrins. *J Cell Sci* **109**: 2161–2168

Hoffman S, Dutton SL, Ernst H, Boakle MK, Everman D, Tourkin A and Loike JD (1994) Functional characterisation of antiadhesion molecules. *Perspect Dev Neurobiol* **2**: 101–110

Langdon SP, Lawrie SS, Hay FG, Hawkes MM, McDonald A, Hayward IP, Schol DJ, Leonard RCF and Smyth JF (1988) Characterization and properties of nine human ovarian adenocarcinoma cell lines. *Cancer Res* **48**: 6166–6172

Mackie EJ, Chiquet-Ehrismann R, Pearson CA, Inaguma Y, Taya K, Kawarada Y and Sakakura T (1987) Tenascin is a stromal marker for epithelial malignancy in the mammary gland. *Proc Natl Acad Sci USA* **84**: 4621–4625

Mould AL, Askari JA, Craig SE, Garratt AN, Clements J and Humphries MJ (1994) Integrin α4β1-mediated melanoma cell adhesion and migration on vascular adhesion molecule-1 (VCAM-1) and the alternatively spliced IIICS region of fibronectin. *J Biol Chem* **269**: 27224–27230

Pearson CA, Pearson D, Shibahara S, Hofsteenge J and Chiquet-Ehrismann R (1988) Tenascin: cDNA cloning and induction by TGF-β. *EMBO J* **7**: 2977–2981

Rettig WJ, Erickson HP, Albino AP and Garin-Chesa P (1994) Induction of human tenascin (neuronectin) by growth factors and cytokines: cell type-specific signals and signalling pathways. *J Cell Sci* **107**: 487–497

Spring J, Beck K and Chiquet-Ehrismann R (1989) Two contrary functions of tenascin: dissection of the active site by recombinant tenascin fragments. *Cell* **59**: 325–334

Walker RA, Daring SJ and Gallacher B (1994) Relationship of transforming growth factor β1 to extracellular matrix and stromal infiltrates in invasive breast carcinoma. *Br J Cancer* **69**: 1160–1165

© Cancer Research Campaign 1999 

Wilson KE, Langdon SP, Lessells AM and Miller WR (1996) Expression of the extracellular matrix protein tenascin in malignant and benign ovarian tumours. *Br J Cancer* **74**, 999–1004

Yokasaki Y and Sheppard D (1995) Integrin receptors for extracellular matrix with special reference to tenascin. *Trends Glycosci Glycotechnol* **7**: 417–427

*© Cancer Research Campaign 1999*
